# Research focusing on plant performance in constructed wetlands and agronomic application of treated wastewater – A set of experimental studies in Sicily (Italy)

**DOI:** 10.1371/journal.pone.0219445

**Published:** 2019-07-09

**Authors:** Mario Licata, Maria Cristina Gennaro, Teresa Tuttolomondo, Claudio Leto, Salvatore La Bella

**Affiliations:** 1 Department of Agricultural, Food and Forest Sciences, Università degli Studi di Palermo, Palermo, Italy; 2 Research Consortium for the Development of Innovative Agro-environmental Systems, Palermo, Italy; Centre for Ecology and Hydrology, UNITED KINGDOM

## Abstract

Constructed wetlands are sustainable technologies for the treatment of wastewater. These biological systems have been widely studied throughout the world for more than 30 years; however, most studies have focused on the effects of design and engineering on pollutant removal from wastewater. Undoubtedly, agro-technical aspects have been given too little consideration by research. This paper reports the main results of a set of experiments carried out on two pilot horizontal subsurface flow systems in Sicily (Italy). *Festuca*, *Lolium* and *Pennisetum* spp. in combination and three emergent macrophytes–*Arundo donax* L., *Cyperus alternifolius* L. and *Typha latifolia* L.–alone, were assessed. The aim of the study was to demonstrate that, under predetermined hydraulic and design conditions, the choice of plant species and the management of the vegetation can significantly affect the pollutant removal performance of constructed wetlands. In addition, wastewater (after treatment) can also be used for agricultural purposes leading to increased sustainability in agricultural systems. *Arundo* and *Typha*-planted units performed better than *Cyperus*-planted units in terms of chemical, physical and microbiological contaminant removal. All the species adapted extremely well to wetland conditions. Polyculture systems were found to be more efficient than monocultures in the removal of dissolved organic compounds. The reuse of treated wastewater for the irrigation of open fields and horticultural crops led to significant savings in the use of freshwater and fertilizers. The results of physical-energy characterization of *A*. *donax* above-ground plant residues and pellets highlighted the fact that a constructed wetland could also be a potential source of bioenergy.

## Introduction

Constructed wetlands (CWs) are artificial ecosystems for the treatment of wastewater and represent an alternative to conventional biological systems. These systems have been of great interest to many countries for a long time, and the horizontal subsurface flow system (HSSFs) is one of the most commonly used systems around the world [1–3]. Plants, substrate and microorganisms are the main components of CWs and their interaction is fundamental for the optimal functioning of the system [4]. Literature on the subject has largely focused on the importance of design and engineering in CWs [5–7] and little attention has been paid to agro-technical related aspects. Relatively few studies report the fact that factors such as the choice of plant species, plant density and cropping systems can significantly affect the performance of CWs in terms of pollutant removal efficiency, greenhouse gas emissions and energy outputs. The choice of plant species is crucial in the design of CWs due to various functions carried out by plants. We know, for example, that the size of the plant roots can affect the hydraulic characteristics of the substrate and increase the retention time of wastewater in the substrate [8]. Literature has also shown that plants promote a number of physical effects, such as filtering, increased rate of sedimentation and reduced risk of re-suspension [9]; the direct effects of oxygen levels (released by the roots into the rhizosphere) on microbial activity and growth [10] is also well-documented. However, plant species differ in their ability to purify wastewater and a number of factors need to be taken into examination before these differences can be clarified. As an example, plants should be selected on the basis of their availability in the surrounding environment, their adaptive capacity to specific climate conditions, their capacity for sexual and/or asexual propagation, their ability to survive in saturated or unsaturated substrate conditions, their growth rate and their competitive ability against weeds [4]. Today, the choice of cropping system for a CW represents a controversial issue amongst researchers, mainly due to the different ways in which monoculture or polyculture affects the system. Polyculture systems seem to be more efficient at removing wastewater pollutants than monoculture systems for a number of reasons. Firstly, the mix of species provides high pollutant removal performance as the various species provide a range of adaptive capacities to changes in wastewater composition over the short and long term. Secondly, a more homogenous distribution of root systems in the rhizosphere leads to the development of biofilms, which influence most of the microbial processes [1, 11–12]. In contrast, monoculture systems affect the rate of plant-cover establishment and the cost of planting, and they do not require an evaluation of inter-specific competition for climate and nutritional factors. Treated wastewater (TWW) and plant biomass are the main outputs of CWs. Many studies carried out in the Mediterranean region argue that TWW is an important source of water and nutrients in the irrigation and fertilization of horticultural and open-field crops [13–20]. Its use can lead to significant savings in freshwater (FW) and mineral fertilizers compared to conventional methods; these benefits are more evident in areas suffering from water scarcity in the spring and summer seasons. However, the amount of TWW available for irrigation greatly depends on the water budget in the system [21]. Evapotranspiration and rainfall are the main components of the water budget and both can significantly affect pollutant removal efficiency: abundant rainfall dilutes pollutant concentrations and decreases hydraulic retention time (HRT), whilst high ET reduces quantities of treated wastewater and increases pollutant rates at the CW outlet [22–23]. How macrophyte species can affect evapotranspiration rates and the final amount of TWW at the CW outlet is, undoubtedly, worthy of investigation. In Mediterranean climates in particular, macrophytes grow intensively during spring and summer. At the beginning of autumn, however, senescence starts and the vegetation begins to decompose, thereby increasing the nutrients and organic matter content in the system. Plants can be harvested in summer, autumn or winter and the choice of harvest time can affect the performance of a CW in terms of pollutant removal efficiency, microbial activity, dissolved oxygen content and the amount of nutrients transferred to ground biomass [24–25]. If the use of biomass is permitted by law, it can be used as fodder for livestock, fertilizer or soil conditioner, but it can also be converted into bioenergy through direct combustion. Preliminary investigation of a number of aspects, such as the availability of plant biomass in the long term and plants energy yields, are essential, however [26]. This paper reports the main results of a set of experiments carried out on two HSSFs CWs in Sicily (Italy) between 2002 and 2016. We tested three emergent macrophytes and two different water sources. We addressed five key questions: *(a)* How the choice of plant species and cropping system affects the performance of a CW; *(b)* How ET affects pollutant removal rates in a CW; *(c)* How TWW irrigation affects the yield and qualitative characteristics of some open-field and horticultural crops; *(d*) If TWW is able to represent a way of saving nutrients and FW, and *(e*) If wetland biomass is able to be exploited for energy purposes.

## Materials and methods

### Experimental CW system

#### HSSFs CW(1)

**Fig 1:**
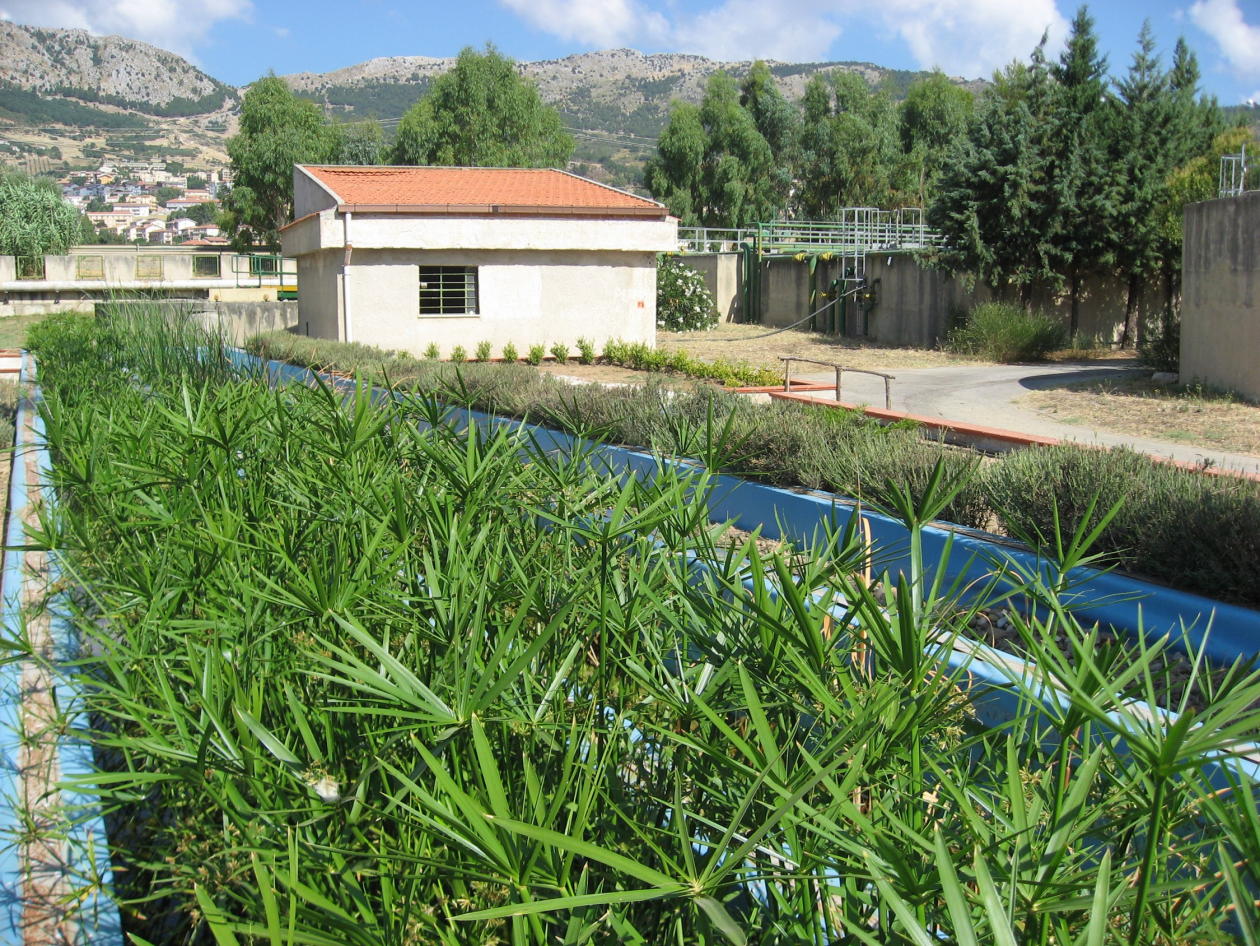
An overview of pilot HSSFs CW(1).

**Fig 2:**
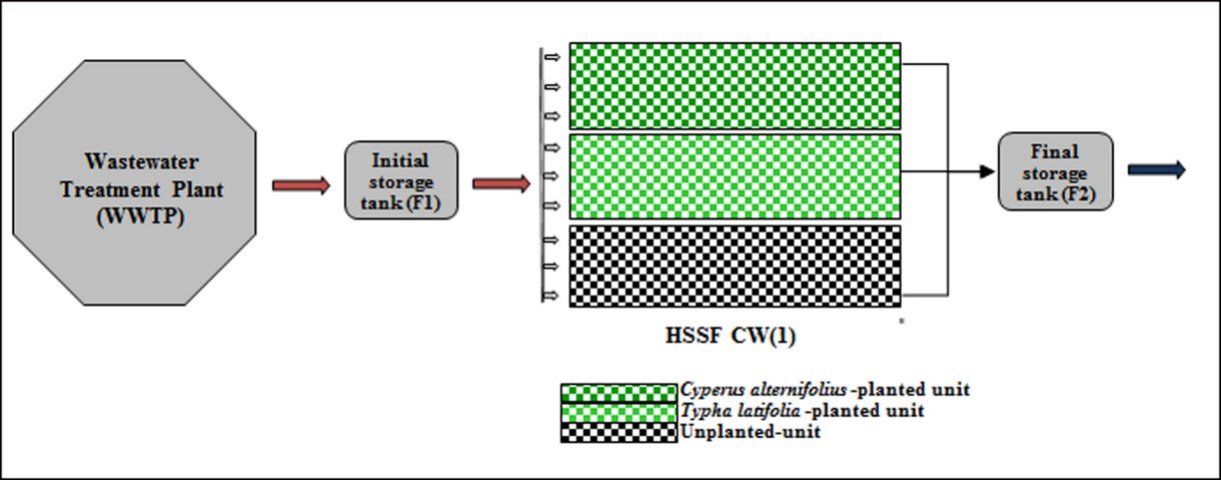
Layout of pilot HSSFs CW(1).

A pilot HSSFs CW(1) was located in Piana degli Albanesi (37°59'56"40 N—13°16'50"16 E, 740 m a.s.l.) in the West of Sicily. This site is characterized by a Mediterranean climate. The average annual temperature is 16°C, with average minimum and maximum temperatures of 10.4°C and 20.2°C. Average annual rainfall is 800 mm. The experimental system was built in 2008 and became operational in 2009. It was situated downhill from the town’s wastewater treatment plant (WWTP) and treated urban wastewater was pumped directly from the WWTP (Fig 1). The system consisted of three independent units lying in parallel (Fig 2), each 33 m long and 1 m wide. The total surface area of the experimental system was 99 m^2^.

In March 2009, two units were separately planted with umbrella sedge (*Cyperus alternifolius* L.) at a density of 5 stems m^-2^, and common cattails (*Typha latifolia* L.) at a density of 4 rhizomes m^-2^, whilst the third unit remained unplanted. A silica quartz river gravel (Si, 30.32%; Al, 5.23%; Fe, 6.87%; Ca, 2.79%; Mg, 1.01%), 20–30 mm in diameter, was used for the tests. The depth of each unit was 0.5 m and the slope was 1.5%. The units were lined with sheets of ethylene and vinyl-acetate and were designed in order to receive a total of 3 m^3^ of wastewater per day.

#### HSSFs CW(2)

**Fig 3:**
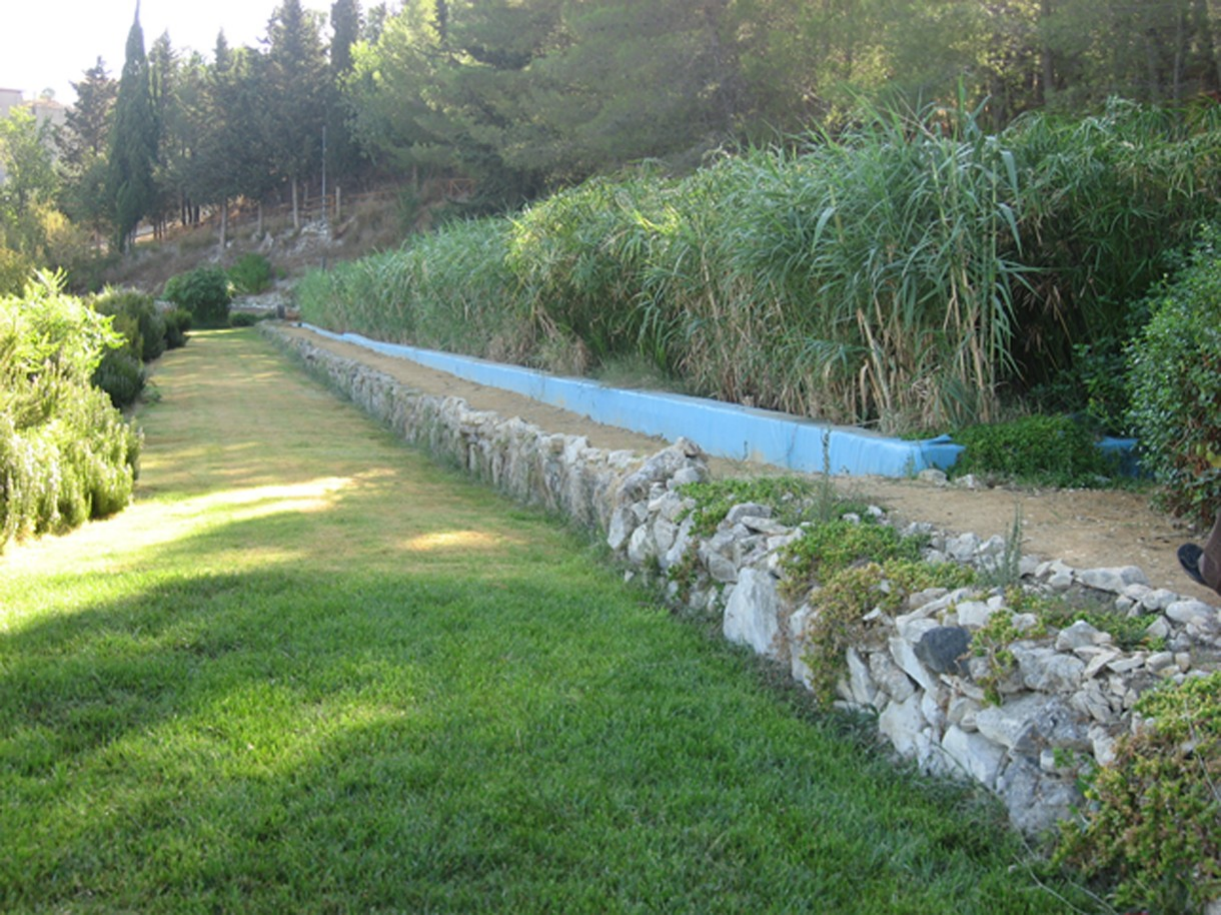
A overview of pilot HSSFs CW(2).

A second pilot HSSFs CW(2) was located in Raffadali (37°24’N– 1°05’E, 446 m a.s.l) in the South-West of Sicily. Raffadali is characterized by a Mediterranean climate. The average annual temperature is 17.5°C, with average minimum and maximum temperatures of 11.2°C and 23.5°C. Average annual rainfall is approximately of 650 mm. The experimental system in Raffadali was located in an open urban park (Fig 3) and was fed with urban wastewater from the WWTP in the town. The main operational parameters are reported in Table 1.

**Table 1:** Main operational parameters of the two pilot HSSFs CWs.

Parameter	unit	HSSFs CW(1)	HSSFs CW(2)
**Inflow rate**	m^3^ d^-1^	3.0	6.0
**Hydraulic loading rate (HLR)**	cm d^-1^	3.0	6.0
**Hydraulic retention time (HRT)**	d	16.5	8.3

**Fig 4:**
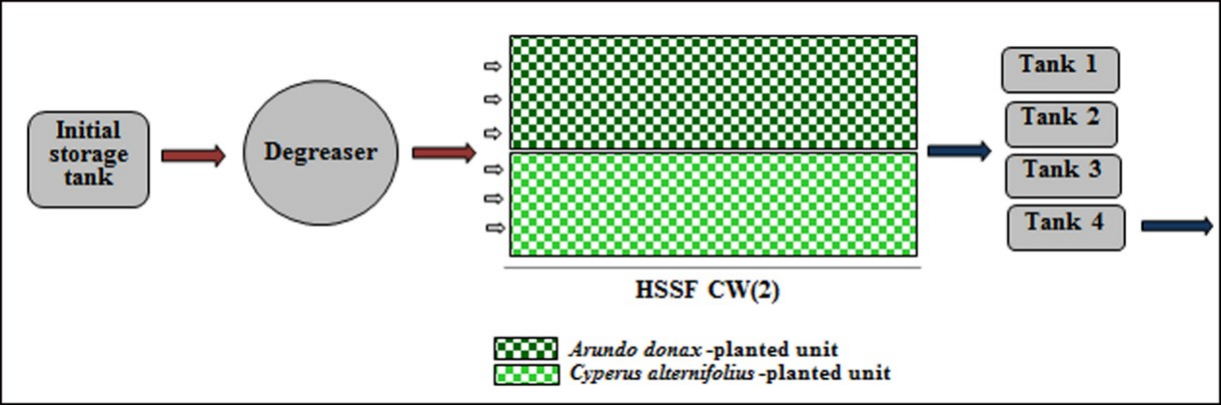
Layout of pilot HSSFs CW(2).

It was built in 1999 and became operational in 2000. The system consisted of two independent units in parallel (Fig 4), each 50 m long and 1 m wide. The total surface area of the experimental system was 100 m^2^.

In February 2000, the two units were initially planted with cool-season species (*Lolium* and *Festuca*), while in July a warm-season species (*Pennisetum*) was planted in the system. In February 2008, the two units were separately planted with giant reed (*Arundo donax* L.), at a density of 4 rhizomes m^-2^, and with umbrella sedge, at a density of 5 stems m^-2^. A silica quartz river gravel (Si, 30.02%; Al, 5.11%; Fe, 6.10%; Ca, 2.65%; Mg, 1.05%), 30 mm in diameter, was used. The depth of each unit was 0.5 m and the slope was 2.0%. The units were lined with sheets of ethylene and vinyl-acetate and were designed in order to receive a total of 6 m^3^ of wastewater per day. In both the experimental systems, the distribution of wastewater was homogenous in the units and the pumping of wastewater was continuous throughout the day without variations in time. The main operational parameters are reported in Table 1.

### Plant growth analysis

Plant height (S1 Table), stem density, fresh and dry weight of the above-ground (leaves and stems) and below-ground (roots and rhizomes) plant parts were considered to analyze plant growth between 2011 and 2013. A representative sample of 10 plants, selected randomly from various sections of each experimental unit for both HSSFs CWs, was used to determine average plant height. Stem density was randomly determined on an area of 1 m^2^ for each unit. Four main crop growth stages [27] were identified. In November of each year, the plants were cut back to a height of 50 cm above gravel surface. The fresh above-ground and below-ground plant parts were determined on a sample of 10 plants selected from each unit. The biomass dry weight was calculated by drying the collected plant material in an oven at 62°C for 72 hours. A carbon, hydrogen and nitrogen (CHN) analyzer was used to measure the nitrogen (N) levels in the above-ground and below-ground plant parts, in accordance with plant biomass basic analysis standards.

### Urban wastewater analysis

In HSSFs CW(1), monthly sampling campaigns were carried out between April-October of each year between 2010–2016, amounting to a total of 288 samples. In HSSFs CW(2), samples were taken in a non-continuous manner between April and October in the years 2002 to 2015; 156 samples were taken in total. In both the HSSFs CWs, the samples were collected at the inlet and outlet of each unit. The sampling of influent and effluent was carried out instantaneously. pH, electrical conductivity (EC), temperature (T) and dissolved oxygen levels (DO) were determined directly on site using a Multiline WTW P4. Total suspended solids (TSS), biochemical oxygen demand (BOD_5_), chemical oxygen demand (COD), total Kjeldahl nitrogen (TKN), ammonia nitrogen (NH_4_-N) and total phosphorus (TP) were determined in the laboratory, using Italian water analytical methods [28]. Total coliform (TC), faecal coliform (FC), faecal streptococci (FS), *Escherichia coli* (*E*. *coli*) and *Salmonella* spp. levels were also measured by membrane filter methods [29]. Removal efficiency (RE) of the two experimental systems was calculated based on pollutant concentrations [30]: $RE\;=\frac{C_{i-}C_{0}}{C_{i}}*100$ where: C_i_ and C_0_ are the mean concentrations of the pollutants in the influent and effluent, respectively.

### Water budget calculation

Daily evapotranspiration (ET_0_) was calculated using the Penman-Monteith equation:

ET_0_ = $\frac{0.408\;∆\;\left(Rn-G\right)+\gamma \left(900/T+273\right))u2(es-ea)\;}{∆+\;\gamma (1+0.34u2)}$ where: R_n_ is net radiation at the crop surface (MJ m^2^ d^-1^)G is soil heat flux density (MJ m^2^ d^-1^), T is average air temperature (°C), u_2_ is wind speed at 2 m height (m s^-1^), e_s_ is the saturation vapour pressure (kPa), e_a_ is the actual vapour pressure (kPa), e_s_- e_a_ is the saturation vapour pressure deficit (kPa), Δ is the slope of the vapour pressure curve (kPa°C^-1^), γ is the psychrometric constant (kPa°C^-1^). From 2013 to 2015, water budget was estimated separately for each unit every 10 days from April to November, taking into consideration the growth stages of the three studied macrophytes. The following equation [30] was then used for calculation: Q_0_ = $\frac{Qi+(P-ETc)\;}{A}$ where: Q_o_ is wastewater outflow rate (m^3^ d^-1^), Q_i_ is wastewater inflow rate (m^3^ d^-1^), P is precipitation rate (mm d^-1^), ET_c_ is crop evapotranspiration (mm d^-1^), and A is wetland top surface area (m^2^). The amount of water at the inlet and outlet of each unit was determined using a volumetric flow meter. Rainfall was determined with a pluviometer. ET_c_ was calculated using the equation: ET_c_ = $Q_{i}+P\;\left(A\right)-Q_{0}$. Crop coefficients (K_c_) were calculated for each growth stage of the plants, using the equation [27, 31]: K_c_ = $\frac{{ET}_{C}}{{ET}_{0}}$. In each planted-unit, water use efficiency (WUE) was also estimated using the ratio between above-ground biomass dry weight produced in a year and the total volume of water lost via evapotranspiration in the same period [32].

### Treated wastewater reuse

#### Open field crops

Two experimental fields of bermudagrass [(*Cynodon dactylon* L. (Pers.)] and seashore paspalum (*Paspalum vaginatum* L.) were set up close to the pilot HSSFs CWs during the period 2013–2016. Seeded and vegetative varieties were used for the tests. The single plot size was 4 m^2^. Experimental fields were equipped with sprinkler irrigation systems and plots were irrigated both with FW and TWW. A conventional nitrogen, phosphorus (P) and potassium (K) fertilization program was used to manage the FW-irrigated plots (200 kg N ha^-1^, 40 kg P_2_O_5_ ha^-1^ and 160 kg K_2_O ha^-1^). Fertilization of the TWW-irrigated plots was carried out taking into consideration N, P and K levels in the TWW in order to satisfy plants nutrient requirements. A split-plot design for a two-factor experiment was used with four replications. The main plot factor was irrigation and the subplot factor was species. Plants were mowed by a helicoidal mower and were maintained at a mowing height of 30–35 mm. No insecticide and fungicide treatments were carried out during the test period. The main morphological, production and qualitative parameters of the plants were determined in full accordance with appropriate references [33–35]. Plant biomass was estimated taking a grass sample randomly from each subplot of each irrigation level in June and September of each year.

#### Horticultural crop

An experimental field of tomato (*Lycopersicon esculentum* Mill.) was prepared in the area of the pilot HSSFs CW(1) in 2015. A single variety of tomato (Incas) was tested. The single plot size was 50 m^2^ with a plant density of 2.2 plants m^-2^. The between-plant distance on the row was 30 cm and the inter-row distance was 150 cm. Four drip irrigation systems were used for the tests and drippers were positioned 30 cm apart in each drip system. Plots were irrigated both with FW and TWW. Irrigation was applied from April to June twice a week for 1 h and from July to September twice a week for 3 h. In FW irrigated-plots, we used 80 kg N ha^-1^, 130 kg P_2_O_5_ ha^-1^ and 120 kg K_2_O ha^-1^ for commonly-used fertilization programs for tomato. In TWW irrigated-plots, we estimated the N, P and K levels supplied by irrigating with TWW. A randomized complete block design was used with three replications to test irrigation with four treatment levels. Traditional pest and weed controls of the tomato were also carried out. Fruits were hand harvested at full red stage of maturity from the first 10-days in August to the third 10-days in September at weekly intervals. The main morphological, productive and qualitative parameters of tomato were determined on a sample of 20 marketable fruits from each plot.

#### Soil analysis

Soil samplings were carried out both before sowing or transplanting and at the time of harvesting, taking into consideration only the topsoil (0.30 m). Soil samples were randomly collected from each plot and successfully analyzed. The main parameters were: pH, electrical conductivity (EC), organic matter (OM), total nitrogen (TKN), total organic carbon (TOC), assimilable phosphorus (P), assimilable potassium (K), active calcareous (active CaCO_3_), magnesium (Mg) and sodium (Na) content. International protocols were used for soil parameter analysis.

### Plant biomass use

In HSSFs CW(2), a physical-energy characterization of above-ground biomass of *A*. *donax* was carried out in 2016. Ash content was determined in accordance with UNI EN 14775:2010 Italian standards [36]. Moisture content of the ash was determined in accordance with UNI EN 14774–2:2010 Italian standards [37]. Heating calorific value (HCV) for the ash-free dry matter was determined in accordance with UNI EN 14918:2010 Italian standards [38]. Above-ground residues of giant reed were subsequently tested for pellet-making and the bulk density and mechanical durability (DU) parameters were determined in accordance with UNI EN 15103:2010 Italian standards [39]. DU was calculated using the equation: $DU\;=\;\frac{MA}{ME}*100$ where: MA is the pellet weight after treatment and ME is the pellet weight before treatment.

### Climatic data

Climatic data were collected from two meteorological stations belonging to the Sicilian Agro-Meteorological Information Service situated close to the pilot HSSFs CWs. The stations were equipped with a MTX datalogger and with various sensors for the measurement of global solar radiation, leaf wetness, rainfall, relative humidity, temperature and wind speed.

### Statistical analyses

Statistical analyses were performed with the package SPSS for Windows (version 17.0, Chicago, USA) and included analysis of variance (ANOVA). The difference between means was carried out using the Tukey test. All the representative values were presented using mean ± standard error calculations.

## Results and discussion

### Experiment 1: Effects of plant species on vegetative growth, yield and nitrogen uptake

**Fig 5:**
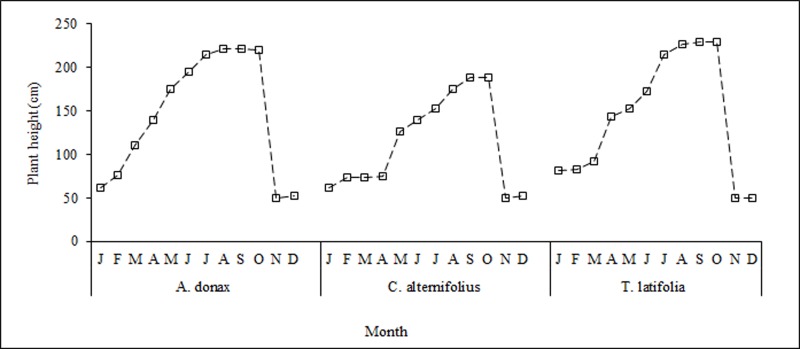
*A*. *donax* L., *C*. *alternifolius* L. and *T*. *latifolia* L. plant height trend. Average values are shown.

During the tests, plant growth increased greatly in summer when global solar radiation, temperature and relative humidity levels were higher (Fig 5).

**Fig 6:**
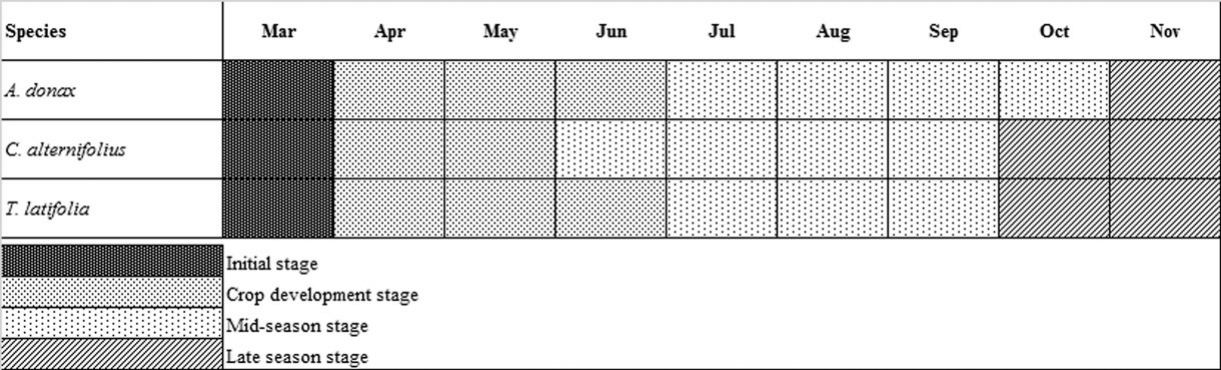
Main phenological stages of *A*. *donax* L., *C*. *alternifolius* and *T*. *latifolia* L. during the test period.

When analyzing the growth stages (S2 Table), considerable differences were found between the species with regard to the duration of each stage (Fig 6).

**Fig 7:**
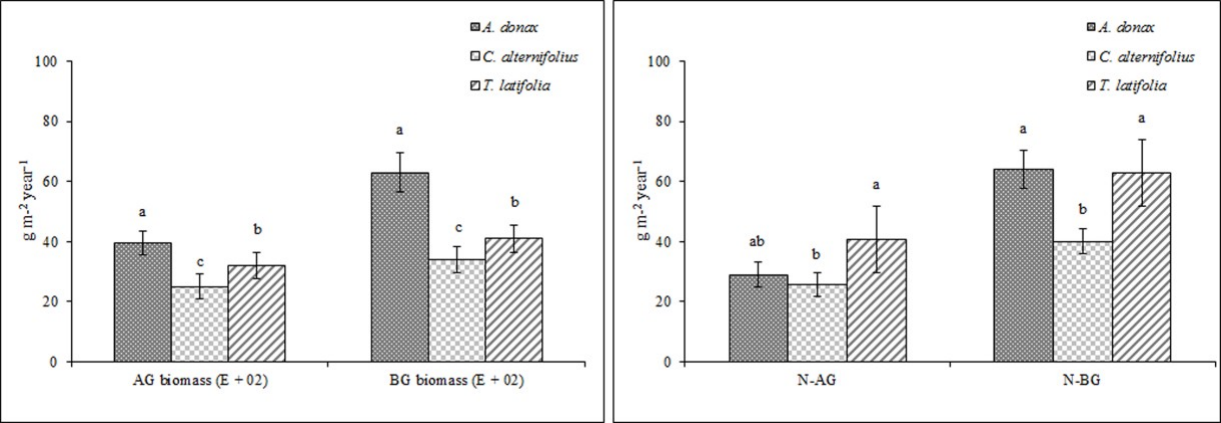
Above-ground (AG) and below-ground (BG) plant biomass production and nitrogen (N) uptake of *A*. *donax* L., *C*. *alternifolius* L. and *T*. *latifolia* L. Average values (± standard error) are shown. Histograms with different letters are significantly different at p ≤ 0.05 and separated using Tukey’s test.

For all the species, vegetative activity decreased at the beginning of winter when minimum and maximum air temperatures fell significantly. Harvesting of the plant biomass was carried out in autumn at the onset of dormancy and when the nutrient uptake capacity of the species slowed down considerably. Vegetative growth began again in spring when air temperatures rose. *A*. *donax* and *T*. *latifolia* produced above- and below-ground biomass levels which were greater on average than those of *C*. *alternifolius* (Fig 7).

When comparing the biomass levels of the three species during the test period (S3 Table), we found considerable differences and this was mainly due to variations in climate conditions and to the different ages of the plants, as the composition of the wastewater was similar throughout the years. Maximum biomass levels were found in the third growth season for *A*. *donax* and *C*. *alternifolius*, and in the fourth for *T*. *latifolia*. On average, the above- and below-ground biomass yields of the three macrophytes were lower than those found in literature, mainly due to the fact that the climate conditions in the study area [40–41] differed considerably to those in literature. In our research, one-way ANOVA showed that average N levels in the above- and below-ground plant parts of *A*. *donax* and *T*. *latifolia* were significantly higher than those of *C*. *alternifolius* (Fig 7).

It is, therefore, reasonable to suppose that greater biomass production levels can favour N uptake and greater nutrient accumulation in the above/below-ground plant parts. This consideration is consistent with the findings of other authors [1–2] when comparing the plant growth rates of various species and using different types of wastewater. These results highlight the fact that both the plant’s ability to adapt to climate conditions and the type of CW system is fundamental in order to obtain high plant biomass levels and, as a consequence, an increase in the pollutant RE of the CW. Furthermore, we can also assume that the same species could perform differently, in terms of biomass yields and pollutant RE, if undergoing changes in climate conditions and/or type of CW system.

### Experiment 2: Effects of cropping system on pollutant removal efficiency

In HSSFs CW(1), when comparing only the planted units, TSS, BOD_5_, COD, TKN, N-NH_4_ and TP effluent concentration rates were lower in the *T*. *latifolia* than in the *C*. *alternifolius-*planted unit (S4 Table). An identical trend was observed in microbiological concentration levels. In fact, both the planted units obtained pathogen levels which were lower than the unplanted unit at the outflow. In addition, when considering only the planted units, FC, TC, FS and *E*. *coli* effluent concentration levels were found to be lower in the *T*. *latifolia* than in the *C*. *alternifolius-*planted-unit. The two planted units had higher RE values than the unplanted control and the *T*. *latifolia*-planted unit had higher RE values than the *C*. *alternifolius-*planted unit for all the chemical and microbiological parameters in the study (Table 2).

**Table 2:** Average (± standard deviation) values pertaining to the main chemical, physical and microbiological water parameters of the influent and effluent of HSSFs CW(1) from 2010 to 2016. Pollutant removal efficiency (%) is also shown.

Parameter	Treatments						
		*T*. *latifolia*		*C*. *alternifolius*		Unplanted	
	Influent	Effluent	RE	Effluent	RE	Effluent	RE
**TSS (mg L**^**-1**^**)**	31.02 ± 4.96	11.13 ± 3.42	64	13.52 ± 3.85	57	23.51 ± 5.23	24
**BOD**_**5**_ **(mg L**^**-1**^**)**	25.32 ± 3.95	8.21 ± 1.83	68	9.25 ± 1.98	64	14.01 ± 3.19	43
**COD (mg L**^**-1**^**)**	54.41 ± 4.70	12.93 ± 3.70	75	15.62 ± 3.96	70	30.91 ± 7.90	51
**TKN (mg L**^**-1**^**)**	18.42 ± 3.30	8.82 ± 1.34	51	10.3 ± 1.51	43	15.2 ± 2.77	17
**NH**_**4**_**-N (mg L**^**-1**^**)**	13.42 ± 1.98	6.62 ± 1.05	52	7.74 ± 1.40	41	10.72 ± 1.39	19
**TP (mg L**^**-1**^**)**	7.82 ± 0.74	4.21 ± 0.71	47	4.91 ± 0.90	38	7.00 ± 0.80	10
**TC (MPN 100 ml**^**-1**^**)**^**^**^	4.37 ± 0.08	3.39 ± 0.17	88	3.54 ± 0.11	85	4.13 ± 0.08	41
**FC (MPN 100 ml**^**-1**^**)**^**^**^	4.24 ± 0.07	3.30 ± 0.16	88	3.45 ± 0.12	83	4.07 ± 0.10	32
**FS (MPN 100 ml**^**-1**^**)**^**^**^	3.91 ± 0.07	3.11 ± 0.03	84	3.27 ± 0.11	77	3.66 ± 0.02	44
***E*. *coli* (CFU 100 ml**^**-1**^**)**^**^**^	3.11 ± 0.06	2.09 ± 0.09	90	2.15 ± 0.08	88	2.82 ± 0.08	48

^^^: the microbiological values are shown as units of Log_10_.

In HSSFs CW(2), TSS, BOD_5_, COD, TKN and TP effluent concentration rates were lower in the *A*. *donax* than in the *C*. *alternifolius-*planted unit. On a microbiological level, the *A*. *donax* had lower pathogen levels than the *C*. *alternifolius*-planted unit at the outflow. Both planted units produced high RE values for all the chemical and microbiological parameters in the study (Table 3).

**Table 3:** Average (± standard deviation) values pertaining to the main chemical, physical and microbiological parameters of the influent and effluent of HSSFs CW(2) from 2009 to 2015. Pollutant removal efficiency (%) is also shown.

Parameter	Treatments				
		*A*. *donax*		*C*. *alternifolius*	
	Influent	Effluent	RE	Effluent	RE
**TSS (mg L**^**-1**^**)**	45.03 ± 10.01	11.45 ± 3.26	74	12.82 ± 3.71	71
**BOD**_**5**_ **(mg L**^**-1**^**)**	31.14 ± 4.88	9.35± 4.23	70	10.98 ± 2.69	64
**COD (mg L**^**-1**^**)**	63.60 ± 8.98	18.03 ± 3.38	71	21.28 ± 3.81	66
**TKN (mg L**^**-1**^**)**	18.20 ± 3.89	9.16 ± 1.86	48	9.91 ± 2.52	45
**TP (mg L**^**-1**^**)**	3.62 ± 0.91	1.83 ± 0.37	48	2.05 ± 0.57	42
**TC (MPN 100 ml**^**-1**^**)**^**^**^	4.49 ± 3.83	3.53 ± 3.05	89	3.65 ± 3.18	85
**FC (MPN 100 ml**^**-1**^**)**^**^**^	4.24 ± 3.44	3.23 ± 3.57	90	3.29 ± 2.75	88
***E*. *coli* (CFU 100 ml**^**-1**^**)**^**^**^	3.05 ± 2.23	2.10 ± 1.54	88	2.22 ± 1.77	85

^^^: the microbiological values are shown as units of Log_10_.

In both of the HSSFs CWs, each planted unit was managed under the same operational parameters (e.g. inflow rate, HLR, HRT) and the three macrophytes grew under the same climate conditions, using the same agronomic practices (e.g. planting date, plant density, harvesting time) and all plants received nutrients from the wastewater. Despite this, we found that the planted units performed differently one to the other in terms of pollutant RE during the test period. This was mainly due to specific ability to adapt to the environment, different establishment times in CWs, diverse levels of tolerance to wastewater composition and competitive ability against weeds. All these factors affected macrophyte growth rates and above-ground and below-ground biomass levels, and contributed to the differing pollutant RE values obtained. Many authors have explained the mechanisms involved in pollutant removal in CWs [1, 4, 5, 8, 9, 42–43] and most of them found considerable differences in pollutant RE between the species regarding one or more pollutants. The reasons for these differences in pollutant RE between species remain mostly unknown. However, these studies suggest that, when comparing two or more species under the same operational conditions, differing pollutant RE values are to be expected. The choice of plant species can significantly affect the performance of CWs and, without doubt, all agronomic practices which can improve the performance of these systems should be implemented and further developed. As an example, changes in plant density in the early stages of establishment of the wetland system can determine variations in pollutant RE values. Vegetation density affects the hydraulic retention time, or rather, HRT increases with increasing plant density [11]. Pollutant RE depends greatly on the contact time between wastewater and below-ground plant parts; therefore, the higher the HRT, the greater the performance of a CW. Further to this discussion, there is currently a lack of consensus amongst researchers on the best cropping system to use in a CW in order to obtain high pollutant RE. In our research, in HSSFs CW(2), we compared a monoculture with a polyculture system at different times, considering two types of wastewater pre-treatment systems. We found different pollutant RE percentage values for dissolved organic compounds, nutrients and pathogens between the two cropping systems (Table 4).

**Table 4:** Pollutant removal efficiency (%) of some chemical and microbiological TWW parameters at the outlet of HSSFs CW(2), when considering monoculture and polyculture systems.

Crop system	Wastewater	CW	Pre-treatment	Parameter						
				TSS	BOD_5_	COD	TKN	TP	FC	*E*. *coli*
**Monoculture system (present study)**	Urban	HSSFs	WWTP	72	67	68	46	45	88	86
**Polyculture system (present study)**	Urban	HSSFs	Degreased Imhoff tank	40	85	n.a.^^^	60	35	85	82
**World-wide systems** **(Vymazal, 2005)**	Urban Agricultural	HSSFs	Mixed systems	83	85	75	42	41	92	n.a.

^^^: not available.

The monoculture system planted with *A*. *donax* performed better in terms of TSS, TP, FC and *E*. *coli* removal efficiencies whilst the polyculture system planted with warm and cool season *Graminae* species was more efficient in terms of dissolved organic compound removal. These results differed from those found by other authors [44–46] who investigated monoculture and polyculture wetlands planted with a number of different plant species (*Canna indica* L., *Cyperus papyrus* L., *P*. *australis* L., *Phalaris arundinacea* L., *T*. *latifolia* L., etc.) over both short and long test periods. In our research, it is evident that the comparison between the systems was made at two different times and using different plant species, therefore, it could be open to criticism. However, it is of interest when considering the effect of cropping systems on pollutant RE. In fact, deciding on the optimal number of species to use is an important aspect of CWs, as the species must survive the potential toxic effects of the wastewater and its variability. Monoculture systems can guarantee high pollutant RE only if the species shows high adaptability to design and engineering features, rapid establishment and fast growth. The systems do not require a preliminary evaluation of the inter-specific competition for nutrients and water, however, it is important to know the competitive ability against weeds. In contrast, in the construction of a polyculture system, it is fundamental that competition levels between species are equal in order to maintain a stable state over time. It may be that a polyculture system is more effective at removing pollutants as the presence of two or more species provides greater wastewater purification action, both spatially and temporally [12]. Furthermore, this type of cropping system can maintain higher pollutant RE over time compared to a monoculture system, coping better with seasonal changes in wastewater composition. This ability is linked to variations in nutrient preferences of the species used in intercropping [20]. Regarding the effect of the two types of cropping system on plant growth, literature shows that monoculture systems can produce more biomass than polyculture systems in the first year, however, in the long term, biomass production is higher in polyculture systems [44–45]. Many studies highlight the benefits of polyculture systems whilst only a few state that monoculture systems are more efficient at nutrient removal [46]. We can only suppose that the choice of cropping system should be based on a variety of aspects, such as the establishment time of plants in the CW, plant growth rate, above- and below-ground biomass levels, competition ability and the stability of the mixed community in the short and long term. Improvements in the landscape, enhancement of habitat for the bacterial community and tolerance to environmental stress are also worthy of consideration in this field of study.

### Experiment 3: Effects of crop evapotranspiration on water budget, water use efficiency and pollutant removal efficiency

**Fig 8:**
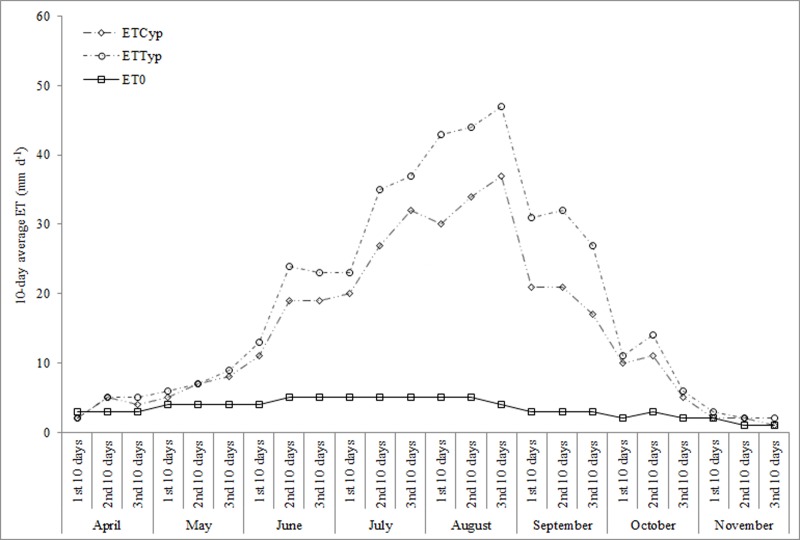
10 day-average ET_0_, ET_typ_ and ET_cyp_ in HSSFs CW(1) during the test period.

**Fig 9:**
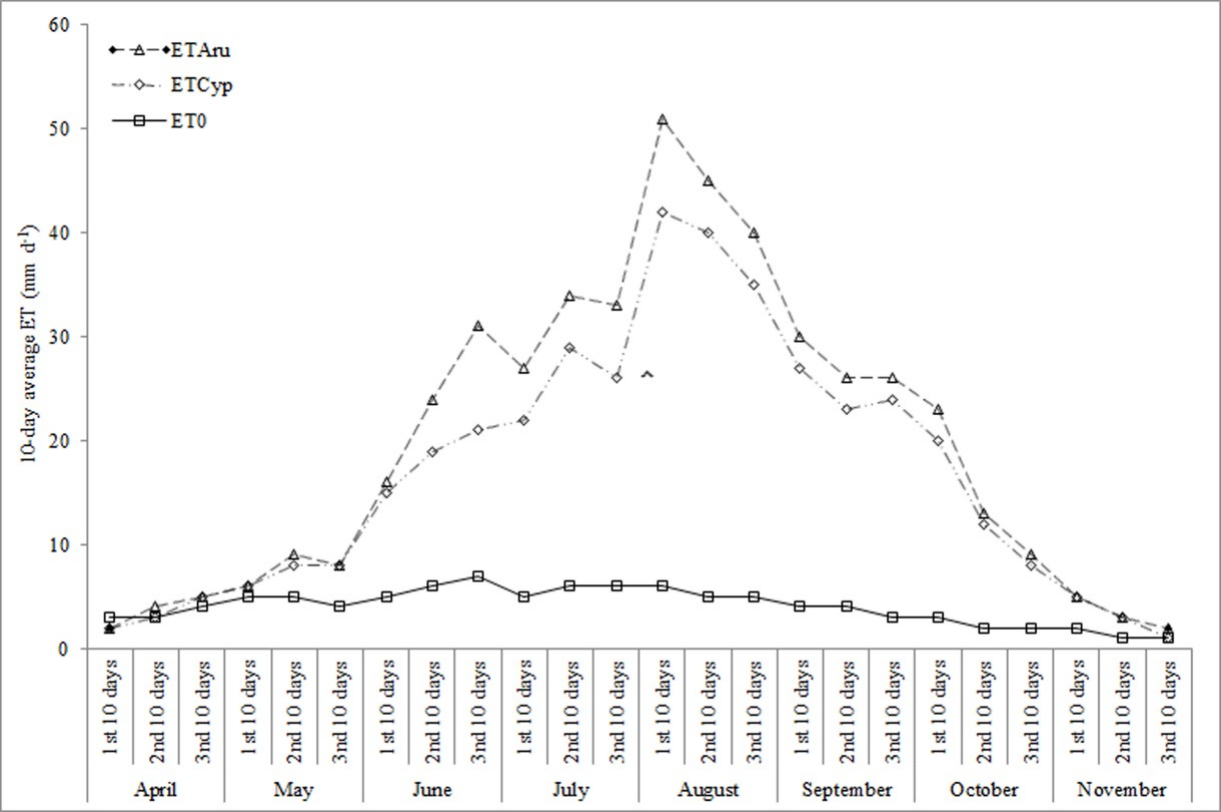
10 day-average ET_0_, ET_aru_ and ET_cyp_ in HSSFs CW(2) during the test period.

Examining results of water budget in both the HSSFs CWs (S6 Table), water loss via ET differed for each of the planted-units. Greater water loss occurred in the *A*. *donax* and *T*. *latifolia*-planted units due to higher growth rate and to greater below-water and above-water plant-part development. It is worth noting that leaf transpiration is higher the wider the leaf, the greater the number of leaves per plant, the taller the plant and the greater the plant density. Based on our findings, we can say that these characteristics were largely found in the *A*. *donax* and *T*. *latifolia*-planted units. From April to November of the test period, average ET_c_ values were found to be higher compared to ET_0_. The *A*. *donax*-planted unit (51.13–2.10 mm d^-1^), *T*. *latifolia*-planted unit (47.02–2.13 mm d^-1^) and *C*. *alternifolius*-planted unit (42.21–1.87 mm d^-1^) were found to have average 10-day ET_c_ values that ranged from a maximum in August to a minimum in November of each year (Figs 8 and 9).

If we consider the growth stages of the three macrophytes, average 10-day ET_c_ values increased during crop development stage, reached a maximum during the mid-season stage and progressively decreased during the late season stage, highlighting a positive relationship between vegetative growth and ET. Similar patterns were found by several authors [23], although using different plant species, adopting different CW systems and carrying out the research under different climate conditions. This highlights the fact that the positive correlation between an increase in plant growth and an increase in ET_c_ occurs only when there are excellent climate conditions for the species selected, independent of the type of system, of the influent wastewater amount and of the operational parameters used. In our study, higher average ET_c_ levels of the planted-units have been explained by the “clothesline” and “oasis” effects [22–23]; however, it is also important to take into consideration the low gravel matrix potential that affects ET rates [47]. With regard to crop coefficient values, they were found to be different for the three macrophytes in the study and varied during each of the growth stages (Table 5).

**Table 5:** Average (± standard deviation) values of crop coefficients of *A*. *donax*, *C*. *alternifolius* and *T*. *latifolia* during the main growth stage in the two HSSFs CWs from 2013 to 2015.

Stage	K_c_		K_c_	
	HSSFs CW(1)		HSSFs CW(2)	
	*C*. *alternifolius*	*T*. *latifolia*	*A*. *donax*	*C*. *alternifolius*
**Initial**	1.05 ± 0.30	1.20 ± 0.34	1.24 ± 0.34	1.08 ± 0.29
**Crop development**	3.39 ± 1.62	3.84 ± 1.79	3.61 ± 1.52	3.21 ± 1.76
**Mid-season**	5.71 ± 0.23	6.51 ± 0.16	7.10 ± 0.12	6.23 ± 0.14
**Late-season**	2.55 ± 1.24	2.95 ± 1.45	3.71 ± 1.61	3.27 ± 1.45

During the three-year tests, higher K_c_ values were recorded for *A*. *donax* and *T*. *latifolia* than *C*. *alternifolius* mainly due to plant growth rates and biomass levels. When comparing average K_c_ values for the three species during main growth stages, we found that the greatest differences were recorded in crop development and mid-season stages. The K_c_ values varied seasonally: the highest values were found during summer, when plant growth increased considerably and the lowest values were recorded during autumn, when plant senescence started. Knowing the K_c_ values of the species during the various growth stages is fundamental for ET_c_ calculation; however, these values are still unknown for a number of emergent macrophytes. Several studies report K_c_ values only for *P*. *australis*, a commonly-used species in HSSFs CWs. When comparing the findings of other authors [48–49], the estimation of K_c_ for *C*. *alternifolius* and *T*. *latifolia* carried out in this study represents new knowledge and these values can be used as a reference to predict ET_c_ in other HSSFs CWs located in areas with similar climate conditions. In this study, the *A*. *donax*- planted unit was found to have higher average WUE values than *C*. *alternifolius* and *T*. *latifolia*. As the wastewater inflow rate was constant for all the 10-day periods in the test, giant reed consumed more water but used water with greater efficiency than common cattails and umbrella sedge, mainly due to greater above-ground dry biomass production (Table 6).

**Table 6:** Average values of TWW outflow rate, above-ground dry biomass and WUE of the *A*. *donax*, *C*. *alternifolius* and *T*. *latifolia*-planted units in the two HSSFs CWs from 2013 to 2015.

Species	Period	Q_0_ (m^3^ 10-d^-1^)	Above-ground dry biomass (g m^-2^)	WUE (g L^-1^)
**HSSFs CW(1)**				
***C*. *alternifolius***	April-November	5.89	2464	0.68
***T*. *latifolia***	April-November	7.05	3210	0.79
**HSSFs CW(2)**				
***A*. *donax***	April-November	8.48	3950	0.93
***C*. *alternifolius***	April-November	4.67	2570	0.64
**HSSFs CW**				
***P*. *australis*** **(Milani and Toscano, 2013)**	April-November	n.a.^^^	9600	2.27

^^^: not available.

**Fig 10:**
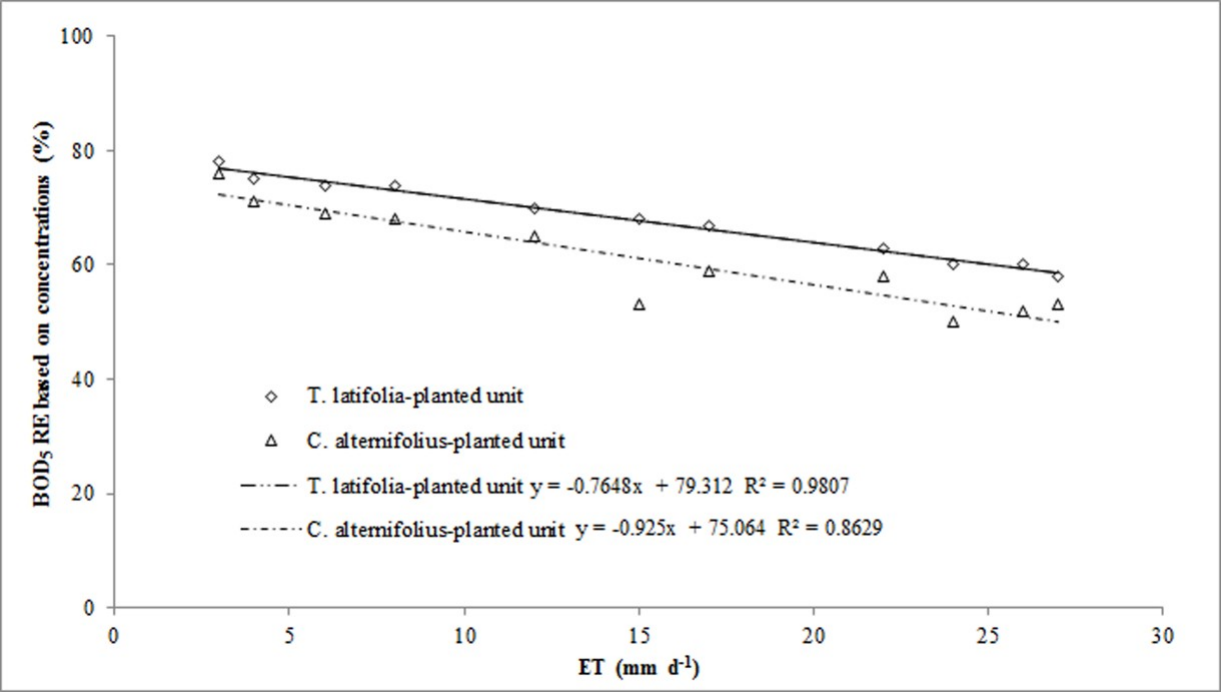
Correlations between evapotranspiration and BOD_5_ RE based on pollutant concentrations in *C*. *alternifolius* and *T*. *latifolia*-planted units.

**Fig 11:**
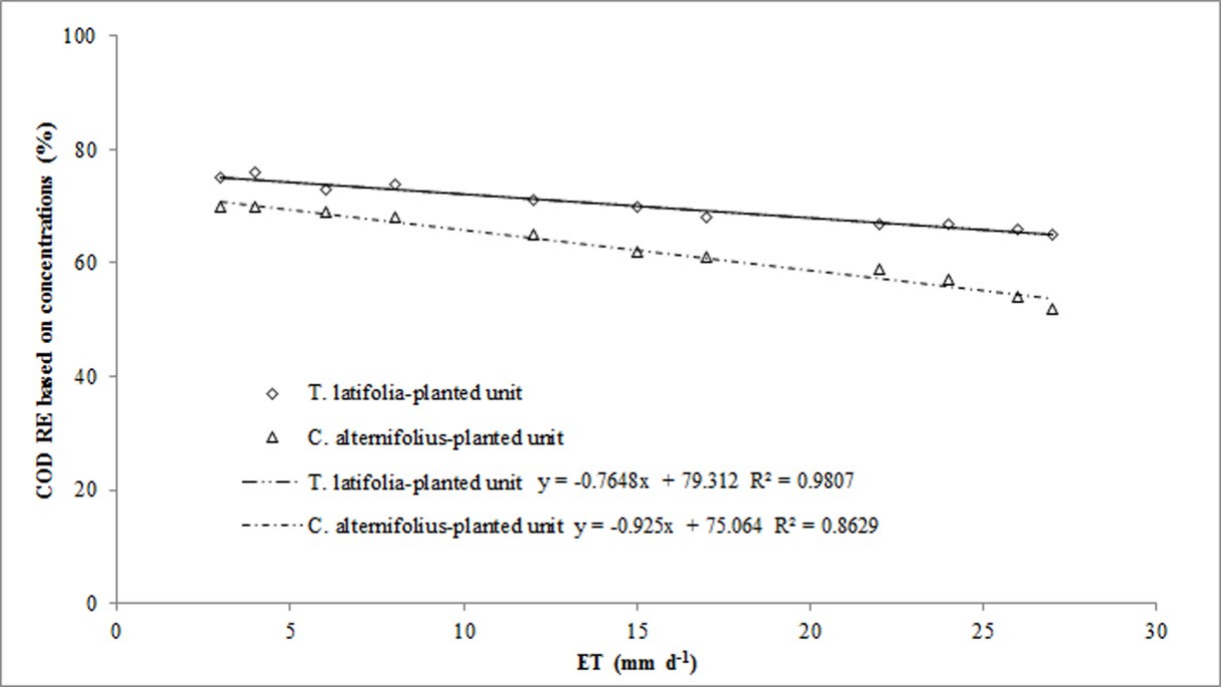
Correlations between evapotranspiration and COD RE based on pollutant concentrations in *C*. *alternifolius* and *T*. *latifolia*-planted units.

We were not able to make a comparison of the three macrophytes with WUE values provided by other studies in the Mediterranean region as no investigation in this field was found. Several studies on *A*. *donax* were carried out on agricultural soils where the plant, soil and water conditions are extremely different to CWs. We thus compared WUE rates with those of *P*. *australis* reported by other authors in a study carried out in Sicily in a HSSFs CW [50]. Here some differences were found due to differing experimental pilot plant size, operational parameters and type of plant species (Table 6). Our results support the theory that increases in above-ground plant biomass over time are directly proportional to increases in WUE. This relationship is highly significant in agronomic terms because it affects the choice of plant species in a CW in relation to our main aims. Some authors [48] claim that ET significantly affects pollutant RE in CWs and high ET values can determine higher pollutant concentrations at the outflow. In our research, in HSSFs CW(1), we found that when ET reached average values of over 20 mm d^-1^, water loss increased at the outlet of the planted-units and we recorded higher BOD_5_ and COD concentrations in the effluent. Analysis of correlation between ET_c_ and RE based on BOD_5_ and COD concentrations showed a decrease in apparent RE of dissolved organic compounds in the planted-units (Figs 10 and 11).

This can be explained when considering the dissolved oxygen concentration levels in the wastewater and how plants and microorganisms use dissolved oxygen and compete for it. In the summer season, competition levels for dissolved oxygen are high, microbial activity tends to fall and, consequently, a decrease in organic compounds RE can be expected. Other authors [47–49, 51] have explained extremely clearly how ET affects pollutant RE in CWs and maintain that, although a relationship is apparent, there is no influence or causation relating to changes in ET on RE. The calculation of pollutant RE can be based either on concentrations or mass loads, but the latter method allows for greater consideration of water flow variations caused by ET. In our research, we calculated BOD_5_ and COD removal efficiencies based on concentrations and mass loads and we were able to estimate how ET affected the amount of water at the outflow (Table 7).

**Table 7:** BOD_5_ and COD concentrations of TWW at the inlet and outlet of HSSFs CW(1) and pollutant RE based on concentrations and mass loads. Average (± standard deviation) values are shown.

Parameters	Treatments					
	*C*. *alternifolius*-planted unit			*T*. *latifolia*-planted unit		
	TWW_(i)_	Concentration RE (%)	Mass load RE (%)	TWW_(i)_	Concentration RE (%)	Mass load RE (%)
**BOD**_**5**_						
**(C)BOD**_**5(i)**_ **(mg L**^**-1**^**)**	26.8 ± 4.5			26.8 ± 4.5		
**(C)BOD**_**5(o)**_ **(mg L**^**-1**^**)**	10.4 ± 2.1	60.5 ± 8.9		9.0 ± 1.1	65.5 ± 7.42	
**(M)BOD**_**5(i)**_ **(g)**	160.7 ± 27.			160.7 ± 27.1		
**(M)BOD**_**5(o)**_ **(g)**	55.4 ± 10.3		65.5 ± 5.5	47.1 ± 4.2		70.7 ± 3.8
**COD**						
**(C)COD**_**(i)**_ **(mg L**^**-1**^**)**	54.5 ± 14.8			54.5 ± 14.8		
**(C)COD**_**(o)**_ **(mg L**^**-1**^**)**	19.1 ± 2.6	63.6 ± 5.9		16.3 ± 2.9	69.3 ± 4.51	
**(M)COD**_**(i)**_ **(g)**	326.7 ± 88.2			326.7 ± 88.7		
**(M)COD (g)**	101.8 ± 15.4		68.8 ± 5.8	84.6 ± 19.5		74.0 ± 1.7
**ET**						
**ET (mm d**^**-1**^**)**	14.2 ± 8.3			16.1 ± 9.4		

(C): concentration; (M): mass load; _(i)_: inlet; _(o)_:outlet.

Data are referred to previous publication [47].

We found that RE of dissolved organic compounds based on mass loads (68.2%) was higher than that based on concentrations (64.9%); however, only during summer months, when ET rates increased greatly. In autumn, when ET decreased quickly, RE of BOD_5_ and COD, calculated with the two methods, was found to be similar (63.1%). Our findings were confirmed by other authors [52], who suggest that RE based on pollutant concentration levels is incomplete because it does not take into consideration variations in water flow due to ET rates.

### Experiment 4: Effects of treated wastewater irrigation on yield and quality of open field and horticultural crops

The chemical and microbiological characteristics of FW and TWW used in this study are shown in Table 8.

**Table 8:** Chemical and microbiological composition of FW and TWW applied to *C*. *dactylon*, *P*. *vaginatum* and *L*. *esculentum* irrigation in the two HSSFs CWs. Average (± standard deviation) values are shown.

Parameter	HSSFs CW(1)			HSSFs CW(2)		
	FW	TWW^a^	TWW^b^	FW	TWW^c^	TWW^d^
**pH**	7.12 ± 0.01	7.32 ± 0.01	7.62 ± 0.03	7.42 ± 0.06	7.12 ± 0.21	7.24 ± 0.91
**EC (μS cm**^**-1**^**)**	299.11 ± 1.82	684.41 ± 5.0	710.11 ± 3.1	301.20 ± 0.62	631.5 ± 11.02	591.5 ± 19.11
**DO (mg L**^**-1**^**)**	n.a.^^^	1.02 ± 0.01	1.10 ± 0.12	n.a.	0.92 ± 0.05	1.01 ± 0.32
**BOD**_**5**_ **(mg L**^**-1**^**)**	1.39 ± 0.41	9.25 ± 1.98	8.21 ± 1.83	1.83 ± 0.11	9.35± 4.23	10.98 ± 2.69
**COD (mg L**^**-1**^**)**	2.32 ± 0.77	15.62 ± 3.96	12.93 ± 3.70	2.39 ± 0.78	18.03 ± 3.38	21.28 ± 3.81
**TSS (mg L**^**-1**^**)**	n.d.^^^^	13.52 ± 3.85	11.13 ± 3.42	n.d.	11.45 ± 3.26	12.82 ± 3.71
**TKN**	n.d.	10.3 ± 1.51	8.82 ± 1.34	2.21 ± 1.01	9.16 ± 1.86	9.91 ± 2.52
**NO**_**3**_**-N (mg L**^**-1**^**)**	0.25 ± 0.15	2.01 ± 0.13	2.21 ± 0.22	0.3 ± 0.12	1.87 ± 0.64	1.99 ± 0.50
**N-NH4 (mg L**^**-1**^**)**	n.d.	7.74 ± 1.40	6.62 ± 1.05	0.11 ± 1.32	5.77 ± 0.12	5.77 ± 0.18
**TP (mg L**^**-1**^**)**	0.60 ± 0.21	4.21 ± 0.71	4.91 ± 0.90	0.87 ± 0.65	1.83 ± 0.37	2.05 ± 0.57
**Ca (mg L**^**-1**^**)**	21.3 ± 0.91	59.11 ± 0.77	56.77 ± 0.55	26.11 ± 0.32	57.03 ± 0.51	60.11 ± 0.58
**K (mg L**^**-1**^**)**	3.41 ± 1.31	73.41 ± 0.69	68.01 ± 0.49	3.01 ± 1.07	67.22 ± 0.37	71.54 ± 1.01
**Mg (mg L**^**-1**^**)**	14.19 ± 1.21	20.22 ± 0.21	21.69 ± 0.29	13.01 ± 1.42	20.5 ± 0.31	20.9 ± 0.22
**Na (mg L**^**-1**^**)**	10.22 ± 0.71	142.12 ± 0.2	134.51 ± 0.6	10.98 ± 0.21	147.8 ± 0.66	152.2 ± 1.71
**TC (MPN 100 ml**^**-1**^**)**	1.19 ± 0.11	3.54 ± 0.11	3.39 ± 0.17	1.01 ± 0.01	3.53 ± 3.05	3.65 ± 3.18
**FC (MPN 100 ml**^**-1**^**)**	1.27 ± 0.31	3.45 ± 0.12	3.30 ± 0.16	1.11 ± 0.03	3.23 ± 3.57	3.29 ± 2.75
**FS (MPN 100 ml**^**-1**^**)**	1.63 ± 0.71	3.27 ± 0.11	3.11 ± 0.03	1.21 ± 0.12	3.11 ± 0.07	3.41 ± 0.12
***E*. *coli* (CFU 100 ml**^**-1**^**)**	1.12 ± 0.31	2.15 ± 0.08	2.09 ± 0.09	0.99 ± 0.03	2.10 ± 1.54	2.22 ± 1.77

^a^: TWW from *C*. *alternifolius*-planted unit;

^b^: TWW from *T*. *latifolia*-planted unit;

^c^: TWW from *A*. *donax*-planted unit;

^d^: TWW from *C*. *alternifolius*-planted unit;

^^^: not available.

^^^^: not detected; the microbiological values are shown as units of Log_10_.

During the test period, TWW from the planted units had higher average values regarding the main chemical and microbiological parameters than FW in both the HSSFs CWs. The lowest variations in nutrient and salt concentrations between TWW and FW were recorded in summer due to higher removal activity of microorganisms and plant uptake. The analysis of the chemical and microbiological characteristics of TWW are important when irrigating open-field crops such as turf species for sport and technical uses. Macro and microelements are, in fact, contained in TWW and affect several physiological processes and plant growth dynamics [53–56]. In our research, significant differences were found between *C*. *dactylon* and *P*. *vaginatum* varieties concerning the morphological and qualitative parameters; however, when comparing the irrigation treatments, FW-irrigated plots and TWW-irrigated plots did not show any significant differences for all the parameters in the study (Table 9).

**Table 9:** Morphological, qualitative and productive characteristics of *C*. *dactylon* and *P*. *vaginatum* varieties irrigated with FW and TWW. Average values are shown.

	Leaf texture (mm)	Horizontal stem density (n cm^-2^)	Visual quality (1–9 scale)	Colour (1–9 scale)	Above-ground dry biomass (kg m^-2^)
**Species**					
***C*. *dactylon***	1.71 A	2.62 B	7.43 B	6.89 B	4.42 B
***P*. *vaginatum***	2.93 B	1.11 A	6.10 A	5.71 A	3.01 A
**Irrigation**					
**FW**	1.98 A	1.84 A	5.77 A	6.11 A	5.67 A
**TWW**^**^**^	2.09 A	1.79 A	6.10 A	6.23 A	5.48 A
**TWW**^**^^**^	2.13 A	1.89 A	5.98 A	6.35 A	6.01 A
**Species x Irrigation**	n.s.	n.s.	*	*	n.s.

Means followed by the same letter are not significantly different according to the Tukey test (P ≤ 0.01).

*: significant, n.s.: not significant.

^^^: TWW irrigation *A*. *donax*-planted unit;

^^^^: TWW irrigation from *C*. *alternifolius*-planted unit.

When considering the Na content, despite higher Na concentration levels in TWW, any aesthetic anomalies or injuries were recorded in TWW-irrigated plants mainly due to short-term application of TWW. Literature [56–57] claims that high Na levels in water can determine macronutrient deficiency, however, as stated by some authors [58], turf species are more tolerant to Na levels than other crops due to the fact they are periodically mowed. However, the effects of Na on physiological processes in plants can be more evident with long-term application of TWW due to increases in Na content in the topsoil and, successively, in the plant tissue. It is important, therefore, to ensure certain agronomic practices are carried out, such as frequent FW irrigation applications for the purposes of Na leaching, in order to avoid excess accumulation of Na in the soil in the long term. In this experiment, the effects of TWW irrigation on turf species in terms of risk to human health from pathogenic bacteria were not determined. In Italy, the reuse of TWW for irrigation purposes is regulated by Ministerial Decree 152/2006. Threshold values pertaining to pathogenic bacteria concentration levels are more restrictive in this regulation compared to those of other nations. During the tests, injury from bacterial activity was observed in the TWW-irrigated plots, however, it is clear that TWW irrigation of *C*. *dactylon* and *P*. *vaginatum* plants should be monitored in the long term in order to assess the probable negative effects of bacteria on the grass and to adopt sustainable solutions to reduce and/or avoid damage. With regard to *L*. *esculentum*, various authors [59–60] have noted that TWW represents a source of nutrients that affects tomato growth, fruit weight, yield and fruit acid concentration, therefore, agriculture could take advantage of this method of using non-conventional water. However, it is crucial to consider the effects of TWW irrigation on the bacterial contamination of the fruits and hazards to human health. Many studies warn of the risks to human health from pathogens entering the food chain following TTW re-use [14, 59]. In our research, TWW-irrigated tomato fruits showed bacteria contamination levels which were higher than FW-irrigated tomato fruits, and concentration levels of most pathogens were not always acceptable in legal terms for crop irrigation. Fruit skin was highly contaminated by FC, FS and *E*. *coli* whilst fruit flesh was uncontaminated [18]. When observing the position of the fruits compared to soil surface, we found that the fruits in contact with the bare soil were more contaminated than others. This was in agreement with previous studies [59–61] that demonstrated the effects of soil moisture from TWW irrigation on bacterial survival and re-growth, the effects of solar radiation and air temperature on microbial contamination of fruit skin, and how fruit skin can represent a favorable habitat for pathogenic bacteria. It is evident that TWW from CWs cannot be considered a source of high-quality water for the irrigation of horticultural crops consumed either raw or cooked, such as tomato. Some authors maintain that an interval between TWW irrigation and fruit harvesting should be given, however, we would recommend the introduction of other disinfection treatments to HSSFs CWs in order to improve the TWW quality in terms of bacteria concentration levels. In our research, when comparing the yields of TWW-irrigated tomato plants with those of FW-irrigated tomato plants, no significant differences were found over the test period (Table 10).

**Table 10:** Effects of the FW and TWW irrigation on yield parameters of the tomato fruits. Average values are shown.

Treatments	Yield parameters				
	Total yield (t ha^-1^)	Marketable yield		Unmarketable yield	
		Total (t ha^-1^)	Per plant (kg plant^-1^)	Total (t ha^-1^)	Per plant (kg plant^-1^)
**FW**	70.51	66.69	2.78	3.82	0.19
**TWW**^**^**^	73.30	69.01	3.56	4.29	0.19
**TWW**^**^^**^	74.01	70.01	2.98	4.00	0.18

No significant differences were observed for each parameter.

^^^: TWW irrigation from *C*. *alternifolius*-planted unit;

^^^^: TWW irrigation from *T*. *latifolia*-planted unit.

These findings on yields were confirmed by a previous study carried out in Sicily [62], although other authors [14] maintain that TWW irrigation determines an increase in marketable yield (MY) compared to FW irrigation. Perhaps it is more correct to say that TWW irrigation can produce both an increase and decrease in MY depending on the different tomato cultivar. This demonstrates that MY is highly affected by genetic factors, even when tomato cultivars are irrigated with the same type of water. When considering qualitative parameters (Table 11), we found some significant differences between TWW and FW-irrigated tomato fruits in terms of pH and SSC.

**Table 11:** Effects of the FW and TWW irrigation on qualitative parameters of the tomato fruits. Average values are shown.

Treatments		Qualitative parameters				
	pH	SSC^a^ (°Brix)	TA^b^ (g 100 ml ^-1^)	DM^c^ (%)	D^d^ (cm)	Co^e^ (a*/b*)
**FW**	4.62 A	4.82 A	0.29 A	5.77 A	1.21 A	2.51 A
**TWW**^**^**^	4.49 B	4.61 B	0.27 A	5.67 A	1.22 A	2.48 A
**TWW**^**^^**^	4.51 B	4.59 B	0.26 A	5.74 A	1.24 A	2.53 A

Means followed by the same letter are not significantly different according to the Tukey test (P ≤ 0.01).

^^^: TWW irrigation from *C*. *alternifolius*-planted unit;

^^^^: TWW irrigation from *T*. *latifolia*-planted unit.

^a^: fruit soluble solids:

^b^: titratable acidity;

^c^: dry matter;

^d^: diameter;

^e^: color.

Our findings were confirmed by other authors [14, 60], who state that the quality of irrigation water can determine significant variations in tomato fruits, especially regarding pH and SSC content, which greatly affect the fruit quality. Moreover, the irrigation treatments did not determine significant differences in diameter and dry matter of the fruits. Regarding the effects of TWW on the chemical characteristics of agricultural soils (S7 Table), we did not find significant differences in pH between FW and TWW-irrigated soils (Table 12). This was probably due to short term application of TWW.

**Table 12:** Effects of the FW and TWW irrigation on soil chemical parameters in the experimental area surrounding HSSFs CW(1). Average values are shown.

Treatments	pH	EC (μ cm^-1^)	TOC (g kg^-1^)	TKN (g kg^-1^)	Assimilable P (mg kg^-1^)	Total CaCO_3_ (g kg^-1^)	Na (ppm)
**FW**	7.64 A	198.11 A	7.45 A	1.15 A	29.35 A	1.27 A	88.50 A
**TWW**^**^**^	7.71 A	231.02 A	10.69 B	1.32 A	39.11 B	1.38 A	95.11 A
**TWW**^**^^**^	7.78 A	229.42 A	10.78 B	1.31 A	42.15 B	1.31 A	92.87 A

Means followed by the same letter are not significantly different according to the Tukey test (P ≤ 0.01).

^^^: TWW irrigation from *C*. *alternifolius*-planted unit;

^^^^: TWW irrigation from *T*. *latifolia*-planted unit.

**Fig 12:**
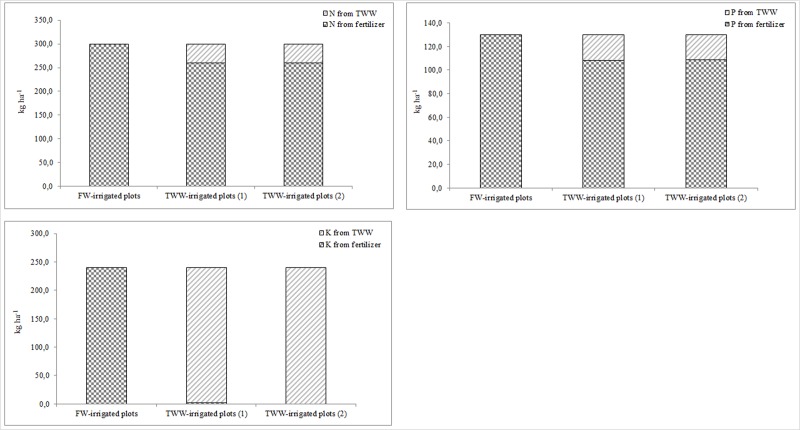
N, P and K fertilizers saving in *C*. *daylon* and *P*. *vaginatum* TWW-irrigated plots compared to FW-irrigated plots.

**Fig 13:**
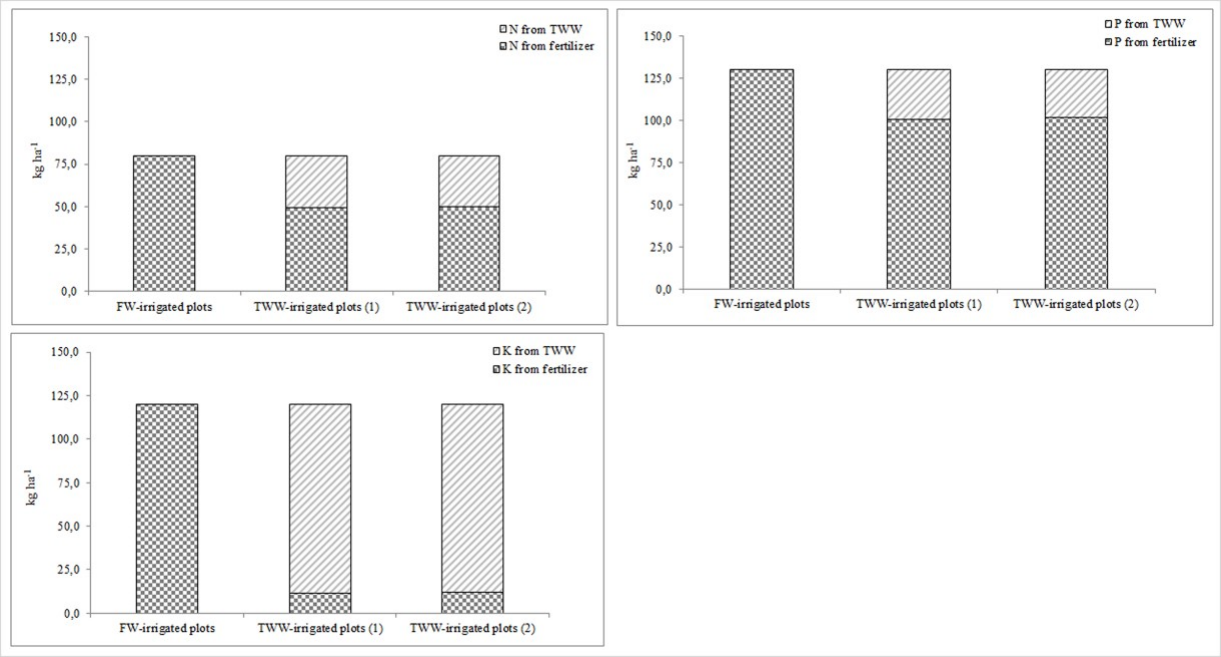
N, P and K fertilizers saving in *L*. *esculentum* TWW-irrigated plots compared to FW-irrigated plots.

During the test period, organic matter content increased in TWW-irrigated soils and this was related to a higher nutrient and organic compound content of TWW compared to FW. No significant differences in salinity were found between the various treatments in the topsoil and this was due to various factors, such as the physical characteristics of the soils (54% sand, 23% silt and 23% clay), the original level of total dissolved salts in the TWW and the short-term application of TWW. Furthermore, we did not observe significant differences in Na content between FW and TWW-irrigated soils, mainly due to low percentages of clay in the soil texture and the occurrence of leaching. Other authors [63] affirm that long-term TWW irrigation can significantly affect certain chemical and physical soil characteristics. Consequently, in the long term, it is crucial to adequately manage TWW irrigation in order to avoid excess accumulation of heavy metals, nutrients and salts in the soil and, at the same time, to exploit increased levels of nutrients and organic compounds in the soil to enhance plant growth. An important result of this research was the sustainable management of N, P and K fertilization in the TWW-irrigated plots for both open-field and horticultural crops (S8 and S9 Tables and Figs 12 and 13).

In conventional agriculture, crop fertilization is usually carried out with the use of considerable amounts of organic and mineral fertilizers related to plant nutrient requirements. Fertilization is an expensive agronomic practice and needs rational management due to the dangers of excess nutrient supplies to crops and the environment. TWW contains a significant amount of nutrients which can be exploited and integrated into traditional crop fertilization programs with inevitable benefits in agronomic, economic and environmental terms. In this study, FW-irrigated plants were managed with a commonly-used fertilization program using granular fertilizers whilst in the TWW-irrigated plots, we exploited the nutrient content in TWW to integrate the N, P and K requirements. N and P mineral fertilizers were also applied from April to June in order to sustain plant growth and this was carried out for all the species in the study. We can say, therefore, that TWW irrigation provided combined fertilization for bermudagrass, seashore paspalum and tomato, and this represented a sustainable way to manage fertilization practices. When considering turf species, savings of 38 kg N ha^-1^, 24 kg P_2_O_5_ ha^-1^ and 235 kg K_2_O ha^-1^ on average were obtained in TWW-irrigated plots in comparison with commonly-used N, P and K fertilization programs. Regarding tomato, TWW irrigation provided savings of 27 kg N ha^-1^, 28 kg P_2_O_5_ ha^-1^ and 110 kg K_2_O ha^-1^ on average. These findings highlight the fact that TWW irrigation can reduce mineral fertilizer needs whilst maintaining high production and quality performance of the crops, in agreement with literature [14, 59–62].

### Experiment 5: Effects of wetland biomass on sustainable production of bioenergy

An aspect that has not been extensively studied in previous years is the use of wetland biomass from vegetation pruning. Literature agrees that the factors which most affect the production and use of plant biomass are related to harvesting time, type of species and wastewater quality. Various studies have focused on the effects of biomass harvesting on CW performance in terms of carbon sequestration, nutrient removal and pathogen control, and have also examined the impacts of harvesting time on biomass yield [24–25, 64]. However, the concept of wetland biomass as a source of bioenergy has rarely been reported in literature and detailed research is lacking [65–66]. Wetland biomass is commonly-used as fodder for livestock, soil conditioner or fertilizer due to its nutrient content, but it could be also harvested for bioenergy production. In a study carried out in China on biofuel production from wetland biomass, the authors [66] explain the main aspects linked to improved bioenergy yields in CWs, such as the use of discharged waste nitrogen, the optimization of hydrologic flow patterns and the selection of productive plant species. When considering plant species, it is necessary to examine the adaptation of plants to environmental conditions, tolerance to wastewater properties, the availability of plant biomass and the energy yield of the plant species [26]. *A*. *donax*, *P*. *australis* and *Typha angustifolia* are considered the highest energy producers in CWs [66]; however, when evaluating the biomass yield, *A*. *donax* is the most high-yielding biomass species of those macrophytes, with an annual biomass yield of 35 t ha^-1^on average, in open field conditions [26, 65, 67]. It is clear from literature that *T*. *latifolia* and *P*. *australis* are the most used and suited species for energy production in the world [66], whilst *A*. *donax* is relatively underused in CWs [26]. We felt it necessary in our study to ensure that a physical-energy characterization of above-ground biomass of the *A*. *donax* plants from HSSFs CW(2) was carried out together with analysis of the physical characteristics of pellets made from above-ground residues (Table 13).

**Table 13:** Main physical and energy parameters of above-ground biomass of *A*. *donax* and physical parameters of pellets. Average (± standard deviation) values are shown.

Reference	Above-ground dry biomass (kg m^-2^)	Moisture (%)	Ash (%)	HCV (MJ kg^-1^)	Bulk density (kg m^-3^)	DU of pellet (%)
**Present study**	3.88 ± 0.87	58.35 ± 0.15	5.97 ± 0.05	14.88 ± 0.04	115.75 ± 1.10	92.33 ± 1.15
**Lewandowski et al. (2003)**	0.31–3.71		4.80–7.80	14.80–18.80	n.a. ^^^	n.a.
**Cosentino et al. (2006)**	1.06–2.21	n.a.	n.a.	n.a.	n.a.	n.a.
**Angelini et al. (2009)**	1.60–5.01	n.a.	n.a.	n.a.	n.a.	n.a.
**Zema et al. (2012)**	2.27–6.70	n.a.	n.a.	17.20–18.91	n.a.	n.a.

^^^: not available.

At the time of harvesting, moisture content was found to be within the range of 40–60%. Ash content ranged from 6.10 to 5.82%, whilst HCV was on average 15 MJ kg^-1^. We were not able to compare our findings with those obtained for *A*. *donax* in similar climate and project conditions due to lack of information. When comparing other studies conducted in different climate conditions and using different plant species, we observed differences in terms of biomass yield, ash content and HCV values. As an example, in a study on bioenergy production potential for above-ground biomass from a subtropical CW, the authors [64] assessed 19 emergent macrophytes (not *A*. *donax*) and reported ash content values that varied between 6.8% (*P*. *australis*) and 18.6% (*Ipomea aquatica*) and HCV values that ranged from 16.3 (*Hygrophilla pogonocalyx*) to 18.6 MJ kg^-1^ (*Miscanthus floridulus*). In the Mediterranean area, several studies were carried out in order to evaluate the effects of TWW irrigation on biomass yield and heating values of *A*. *donax* plants grown in agricultural soils [68–70]. It is evident that it is not possible to compare our findings with those provided by those authors, as the results were obtained in different plant growth and cultivation conditions. Furthermore, we used an inert substrate as growth medium and urban wastewater as a source of plant nutrients. However, previous findings (obtained in agricultural soils) can be used as references in order to understand how nutrient rates in TWW affect the energy potential of *A*. *donax* biomass in a CW. Various authors [71] report that greater availability of nutrients in fertilized crops could determine higher biomass yields, facilitate the reduction of ash content and improve biomass combustion. As a result, in a CW, the higher N, P and K concentration levels in the wastewater could determine an increase in plant biomass yield and a decrease in ash content, despite continuous water flow in the system. In our research, the HCV values were on average lower than those provided by other studies carried out in different cultivation conditions and with the use of high fertilization and irrigation inputs [68–70, 72]. In Table 13, we report some physical and energy parameters of above-ground biomass of *A*. *donax* plants grown as an open-field crop. With regard to pellets made from above-ground *A*. *donax* residues, bulk density was on average 115.75 kg m^-3^; this agreed with findings obtained by other authors [73], who evaluated the combustion process of four perennial species grown using different cultivation practices. Mechanical durability of the pellet was on average very high. Our findings support the notion that CWs could also be considered a potential bioenergy source, helping to provide energy for communities in rural and urban areas; however, a preliminary estimation of wetland biomass yields is required together with an assessment of the physical and energy characteristics of the biomass.

## Conclusions

If we return to the original key-questions: (a) How the choice of plant species and cropping system affects the performance of a constructed wetland: the selection of plant species is affected by the availability of plants in surrounding areas, their ability to adapt to climate conditions, speed of establishment and plant growth in wetland conditions, and competitive ability against weeds. Both monoculture and polyculture systems can guarantee high pollutant RE in a CW but their impact depends on various aspects, such as plant growth rates and the intra- and inter-specific competition levels between the species. Therefore, the different efficiency rates of the two systems must be related to different conditions. (b) How evapotranspiration affects pollutant removal rates in a constructed wetland: ET affects pollutant treatment efficiency in a CW by decreasing water volume and increasing concentration levels of dissolved organic compounds in the TWW, especially when air temperatures are high. Removal efficiency is usually based on initial and final pollutant concentrations; however, RE should be calculated also by taking mass loads of organic pollutants into consideration in order to evaluate the effects of variations in water flow due to the ET rate. (c) How treated wastewater irrigation affects the yield and qualitative characteristics of some open-field and horticultural crops and (d) if treated wastewater can represent a way to save nutrients and freshwater: in arid and semi-arid regions, TWW from CWs represents a source of water and nutrients both for crop irrigation and fertilization and leads to savings in FW and mineral fertilizer consumption in comparison with traditional agronomic practices. However, it is crucial to assess the effects of TWW irrigation on crop production in the long term from the accumulation of salts and sodium in the soil. Furthermore, periodic analysis of pathogenic microorganism contamination levels in the vegetables is essential in both the short and long term, especially in horticultural crops such as tomato. TWW-irrigated vegetables must also be disinfected and cooked before being consumed in order to reduce risk to human health. (e) If wetland biomass can be exploited for energy purposes: wetland biomass can be used for energy purposes and this represents an opportunity to obtain bioenergy from a CW. However, three aspects need to be considered: biomass yields, energy yields and biomass characteristics. In this research, we evaluated these aspects for *A*. *donax* and, on the basis of preliminary findings, results regarding residue and pellet production would seem be of interest. However, research is needed on CW biomass yields in relation to production costs and the energy needs of a community in order to evaluate its contribution as a useful sources of energy.

## Supporting information

S1 Table Main average dataset of plant height for the analyses. (XLS)

S2 Table Number of days of each phenological stage for the analyses. (XLSX)

S3 Table Main average dataset of above and below-ground biomass and nitrogen content for the analyses. (XLSX)

S4 Table Main average dataset of the TWW chemical and microbiological parameters of HSSF CW(1) for the analyses. (XLS)

S5 Table Main dataset of climate data in the two experimental sites. (XLSX)

S6 Table Main 10-day average dataset of ET_c_, Q_i_ and Q_0_ in the two pilot HSSF CWs for the analyses. (XLSX)

S7 Table Main 3-year average dataset related to pH, organic compounds, nutrients and salts content in FW and TWW-irrigated soils. (XLS)

S8 Table Management of N, P and K fertilization programs for turfgrass plots irrigated with FW and TWW. (XLSX)

S9 Table Management of N, P and K fertilization programs for tomato FW-irrigated plots and TWW-irrigated plots. (XLS)

S10 Table Main average dataset related to yield parameters of tomato plants irrigated with FW and TWW. (XLSX)
